# Association of Body Mass Index and Waist Circumference with Periodontal Disease

**DOI:** 10.3290/j.ohpd.c_2017

**Published:** 2025-06-03

**Authors:** Bogeun Lee, Sojung Mun

**Affiliations:** a Bogeun Lee Department of Dental Hygiene, College of Software and Digital Healthcare Convergence, Yonsei University, South Korea. Contributed to the conception and design; collected data; conducted the data analysis; drafted and approved the final draft of the submitted manuscript.; b Sojung Mun Associate Professor, Department of Dental Hygiene, College of Software and Digital Healthcare Convergence, Yonsei University, Wonju, 26493, South Korea. Conceived, researched and conducted study; wrote and reviewed manuscript. Contributed to the conception and design; collected data; conducted the data analysis; drafted and approved the final draft of the submitted manuscript.

**Keywords:** body mass index, waist circumference, periodontal disease, nutrition survey, obesity

## Abstract

**Purpose:**

Obesity results in many chronic diseases, and appropriate measurement of obesity will accurately evaluate the risks of other diseases. Studies have primarily focused on the correlation between a single obesity index and periodontal diseases, and studies analysing the correlation between obesity and periodontal diseases using two or more obesity indices are scarce. This study was designed to evaluate the risk of periodontal disease by combining body mass index (BMI) and waist circumference (WC).

**Materials and Methods:**

We analysed BMI and WC of 12,689 adults who participated in the Korea National Health and Nutrition Survey from 2016 to 2018. Participants’ general characteristics included gender, age, marital status, education, income level, smoking, alcohol use, physical activity, oral health examination, tooth brushing, diabetes, hypertension, and dyslipidemia. periodontal diseases were determined using the Community Periodontal Index (CPI). BMI and WC were used as obesity indices. BMI was classified into underweight, normal, and high; WC was classified into normal and high. Based on the classifications, participants were categorised into six levels of obesity.

**Results:**

The risk of periodontal disease was higher in groups 4 (odds ratio [OR]: 2.88; [95% confidence interval [95% CI]: 2.16–4.04]) and Group 6 (OR: 2.91; 95% CI: 2.22–3.83) where WC was high than in Group 5 (OR: 1.79; 95% CI: 1.34–2.40), where BMI was high.

**Conclusion:**

The prevalence of periodontal disease is higher among obese WC subjects. High WC could be a potential risk factor for periodontal disease in adults.

Obesity is an important public health issue globally, causing geriatric and other chronic diseases.^2,5^ Obesity also increases the risk of coronary artery disease, hypertension, diabetes, and other systemic diseases and negatively affects the psychological, emotional, and social aspects of health in individuals. According to the 2017–2018 American National Health and Nutrition Examination Survey (NHANES), the prevalence of obesity in the American adult population is 42.5%.^4^ Similarly, the prevalence of obesity in the South Korean adult population is increasing, from 32.8% in 2015 to 34.6% in 2018.^19^ The World Health Organization (WHO) reported in 2020 that cardiovascular diseases are the leading cause of death, accounting for 55% of deaths. However, the direct link between obesity and cardiovascular diseases remains unclear.^33^ A recent cohort study reported a higher prevalence of cardiovascular diseases associated with a greater degree of obesity. In addition, a meta-analysis on obesity and mortality rates has also reported early deaths in obese patients, suggesting a direct impact of obesity on health risks.^8^

Periodontal diseases are diseases in which bacteria-induced inflammation causes damage to the teeth and periodontal tissues, and are major causes of tooth loss. A recent study found an association between obesity and oral diseases, particularly adult periodontitis.^17^ Moreover, adults with obesity are at an increased risk of metabolic disorders. Obesity triggers the release of adipocytokines, bioactive substances found in fat. These adipocytokines promote inflammation, such as the production of interleukin-6, which weakens the immune system and contributes to periodontal diseases. The mechanism of inflammation in periodontal disease and obesity is that obesity impairs immunological dysregulation and increases blood sugar levels due to acute-phase proteins and proinflammatory cytokines, which also affect periodontal disease.^24^ Additionally, recent studies have reported that Lipopolysaccharide is a virulence factor of Gram-negative bacteria with a crucial importance to the bacterial surface integrity, and is an important activator of innate and adaptive immune responses and tissue destruction cascade. The toxic factors of lipopolysaccharides induce local and systemic endotoxemia, which is considered a risk factor for obesity and diabetes, and the abundant Gram-negative bacteria in lipopolysaccharides act as an important factor in the development of periodontitis.^26^

Therefore, when lipopolysaccharides move into the bloodstream, endotoxemia occurs, and it is possible that they can enter the bloodstream through inflamed periodontal tissues even after a gentle mastication or brushing after dental treatment. This may occur when bacteria move into the systemic circulation after brushing or periodontal treatment, which may support the correlation between periodontitis and systemic diseases due to endotoxemia. Lipopolysaccharide may enter the bloodstream through inflamed periodontal tissues, especially after dental treatment, and even after gentle mastication or tooth brushing.^29^

Thus, recent studies have shown that periodontal disease is not a separate disease independent of systemic diseases, but is related to the comprehensive inflammatory state of the individual, so periodontal disease should also be considered a systemic disease, and periodontal disease affects the susceptibility and progression of systemic diseases, and the reverse interaction should also be considered, so that the systemic inflammatory state can affect the initiation and progression of periodontal disease.^31^ In other words, various inflammatory pathways originating from pathophysiological processes related to many noncommunicable chronic diseases support the reason for the association between periodontitis and chronic noncommunicable diseases.^4,21^ Further, obesity can elevate the risk of periodontal diseases, and conversely, periodontal inflammation can intensify the risk for metabolic disorders, which are linked to obesity.^6^ Moreover, physical inactivity and overeating have been identified as potential risk factors for periodontal diseases. Regular physical activity has been shown to reduce inflammatory processes, which play a critical role in the progression of periodontitis. This reduction in inflammation is supported by studies demonstrating a clear association between physical activity levels and decreased plasma concentrations of inflammatory markers. These findings suggest that engaging in physical activity may help mitigate the risk of developing periodontal diseases by controlling systemic inflammation.^23,27^

Body mass index (BMI) is a commonly used indicator of obesity in clinical practice. BMI is calculated as body weight (kg) divided by height squared (m^2^); However, it does not consider body fat versus muscle composition.^10,32^ For instance, as Asians tend to have a higher percentage of body fat than Westerners for the same BMI, there is a limitation in defining obesity solely based on BMI. Based on the NHANES data, one-third of the population with normal BMI was reported to have abdominal obesity; therefore, it is necessary to consider additional indicators that can complement BMI to provide accurate data for obesity.^7,14^

Waist circumference (WC) is an indicator of the risks associated with excessive abdominal fat. It is measured by locating the upper hipbone and the lowest rib, then placing the measuring tape horizontally around the abdomen between these two points. Abdominal fat contains a high amount of visceral fat, which is formed in the liver, is converted into cholesterol, and released into the bloodstream. This process leads to the accumulation of fat on artery walls, contributing to elevated cholesterol levels, high blood pressure, and cardiovascular diseases.^3^ Similar to BMI, abdominal obesity is a major risk factor of systemic diseases, and the greater the WC, the greater body fat mass and visceral fat, which increases inflammation and thus increases the risk for periodontal disease.^13^ The WHO has acknowledged abdominal obesity as a supplementary indicator of BMI to assess obesity.^18^ Abdominal obesity can be quantified and evaluated through computed tomography (CT) scans or magnetic resonance imaging; however, this equipment is limited and expensive.^15^ So, more straightforward methods, such as measuring the WC, waist–hip ratio, and weight–height ratio, are commonly used. WC has a notable relationship with abdominal fat, especially visceral fat; it accurately accounts for fat distribution in the body and highly correlates with the visceral fat measured through imaging. Therefore, an increasing number of studies on abdominal obesity have used WC as a measurement parameter.^14^ The British National Institute for Health and Clinical Excellence and European WHO Regional Office recommend using both BMI and WC in obesity screening for adults.^19^ A representative indicator of obesity is BMI; however, BMI has limitations in its accuracy, as it cannot differentiate whether the weight is composed of fat or muscle. Additionally, while the relationship between obesity indicators and periodontal disease has been widely reported in the literature, most studies have utilised only a single indicator of obesity. One recent study reclassified obesity based on both BMI and WC and analysed the association between obesity and tooth loss. However, it remains difficult to establish a direct association between obesity and periodontal diseases, as there are many causes of tooth loss besides periodontal disease. Therefore, this study aims to investigate the relationship between obesity and periodontal disease in Koreans by examining the risk of periodontal disease in adults who are categorised as obese either based on BMI or WC individually, or both combined.^19^

## MATERIALS AND METHODS

### Participants

This study utilised data from the Seventh Korea National Health and Nutrition Examination Survey (KNHANES VII). The KNHANES, a national representative survey designed to investigate health and nutritional status, is conducted by the Korean Centres for Disease Control and Prevention (KCDC). Since the KNHANES data are based on multi-stage stratified colony sampling, the analysis was performed considering the stratification variable (Strata: Kstrata), the colony variable cluster: PSU (primary sampling unit), and weight (Wt_oe). Among the 16,486 participants with oral examination results dated from 2016 to 2018, 12,689 adults aged ≥  19 years who had undergone both an obesity test and an oral health survey were included in this study. Disagreement in the total frequency in the results is attributable to omissions due to missing values. Data on the participants’ gender, age, marital status, education, income level, smoking, alcohol use, physical activity (rate of aerobic exercise), oral health examination, tooth brushing, diabetes, hypertension, and dyslipidaemia, BMI, and WC were collected from the health questionnaire, and periodontal diseases status was determined by an oral examination by a public dentist.

The KNHANES received approval from the Institutional Review Board of the KCDC. All the participants in the survey provided written informed consent. Before analysing the data from the KNHANES, the study was approved by the Institutional Review Board of OOOOOO University (CR320128).

### Study Variables

#### Demographic characteristics

We included sociodemographic factors (gender, age, marital status, education level, income level), oral health behaviour (oral examination within 1 year, tooth brushing frequency per day) and health behaviour (alcohol use, smoking, physical activity) and systemic disease (diabetes, hypertension, dyslipidaemia) that may affect periodontal disease as confounding variables. Age groups were 19–34 years, 35–59 years, and  ≥ 60 years.

Smoking status was classified into current smoking status, alcohol consumption status was divided into Lifetime drinking experience, Physical activity was divided into two categories to the aerobic physical activity(at least an average of 2 hours 30 minutes per week of moderate or 1 hours 15 minutes vigorous intensity or mix of moderate-to-vigorous intensity with an amount of time for each activity), systemic diseases (diabetes, hypertension, dyslipidaemia) were classified according to the presence of the disease.

#### Periodontal diseases

The presence of periodontal diseases was evaluated using the Community Periodontal Index based on the WHO standards. The teeth subjected to evaluation included: #11, #16, #17, #26, #27, #31, #36, #37, #46, and #47 when divided into six parts. The criteria for classifying the periodontal tissues were Code 0 for healthy periodontal tissue, Code 1 for bleeding after probing, Code 2 for calculus-forming periodontal tissue, Code 3 for shallow 4–5 mm pockets, and Code 4 for pockets  ≥ 6 mm. Finding  ≥ 4 mm periodontal pockets (Codes 3 and Code 4) in more than one of the six parts was defined as periodontal disease, and Codes 0–2 were defined as normal.

#### Obesity

The height, weight, and WC were measured during the examination, and BMI was calculated as weight (kg)/height squared (m^2^). According to the WHO West Pacific Region and Korean Society for the Study of Obesity guidelines, the degree of BMI was divided into three categories: low (<18.5 kg/m^2^), normal (18.5–25 kg/m^2^), high (> 25 kg/m^2^).^22^ A WC of <90 cm and 85 cm was used as a standard for men and women, respectively, according to the guidelines of the 2005 American Heart Association/National Heart, Lung, and Blood Institute and 2006 Korean Society for the Study of Obesity and the Heart Association and National (Table 1).^16,20^

**Table 1:** Classification of the groups as normal and obese based on the BMI and WC

Group	BMI	WC
Group 1^a^	Underweight	Normal
Group 2^b^	Underweight	High
Group 3^c^	Normal	Normal
Group 4^d^	Normal	High
Group 5^e^	High	Normal
Group 6^f^	High	High
^a^Group 1: Underweight BMI and normal WC, ^b^Group 2: Underweight BMI and high WC, ^c^Group 3: Normal BMI and normal WC, ^d^Group 4: Normal BMI, high WC, ^e^Group 5: High BMI, normal WC, ^f^Group 6: high BMI and WC. BMI, body mass index; WC, waist circumference.

#### Statistical analyses

To analyse the presence of periodontal diseases according to demographic characteristics, a complex sample Chi-square test was performed, and logistic regression was conducted to identify the risk for periodontal diseases based on BMI and WC. Data were analysed using SPSS software version 25.0 (IBM SPSS, Armonk, NY, USA), and a P value of < 0.05 was defined as statistically significant.

## RESULTS

### Prevalence of Periodontal Disease According to Demographic Characteristics

The prevalence of periodontal disease was higher in male participants and in older participants; in participants who did not have annual dental check-ups or did not brush their teeth each day; in participants with diabetes, hypertension, or hyperlipidaemia; and in participants who were obese based on either their BMI or WC (P < .001) (Table 2).

**Table 2:** Crude association between patient characteristics and periodontitis

Characteristics	Division	Periodontal diseases	P value
No	Yes
Gender	Male	3,417 (62.3)	2,128 (37.7)	< .001
Female	5,291 (74.7)	1,853 (25.3)
Age	18–34	941 (2286)	148 (5.9)	< .001
35–59	4,255 (70.4)	1,859 (29.6)
≥ 60	2,167 (53.0)	1,974 (47.0)
Marital status	Married	6,750 (65.1)	3,758 (34.9)
Single	1,957 (90.0)	223 (10.0)
Education	≤ Elementary	1,172 (50.7)	1,147 (49.3)	< .001
Middle	654 (54.9)	522 (45.1)
High	2,827 (72.4)	1,134 (27.6)
≥ College	3,686 (80.0)	968 (20.0)
Income level	Low	2,052 (65.7)	1,092 (34.3)	< .001
Middle–low	2,154 (68.2)	1,043 (31.8)
Middle–high	2,232 (71.2)	959 (28.8)
High	2,252 (72.8)	812 (27.2)
Smoking	Non-smokers	4,071 (78.6)	1,145 (21.4)	
smokers	3,407 (62.3)	2,122 (37.7)
Alcohol use	No	847 (61.6)	520 (38.4)	
Yes	7,794 (70.3)	3,423 (29.7)
Physical activity	No	4,500 (66.0)	2,352 (34.0)	
Yes	3,832 (74.1)	1,411 (25.9)
Oral examination Within 1 year	No	5,314 (66.8)	2,714 (33.2)	< .001
Yes	3,321 (74.0)	1,222 (26.0)
Toothbrushing/day	≤ 1	611 (56.1)	470 (43.9)	< .001
2	3,196 (66.4)	1,660 (33.6)
≥ 3	4,900 (73.6)	1,850 (26.4)
Diabetes	No	8,151 (71.6)	3,363 (28.4)	< .001
	Yes	554 (47.7)	616 (52.3)
Hypertension	No	7,244 (74.2)	2,615 (25.8)	< .001
Yes	1,463 (52.2)	1,365 (47.8)
Dyslipidaemia	No	7,621 (71.4)	3,205 (28.6)	< .001
Yes	1,086 (58.5)	774 (41.5)
Body mass index	Underweight	376 (82.0)	94 (18.0)	
Normal	5,467 (71.7)	2,203 (28.3)	< .001
Obese	2,760 (64.1)	1,628 (35.9)
Waist circumference	Normal	6,434 (72.9)	2,472 (27.1)	< .001
Obese	2,242 (61.0)	1,499 (39.0)	
By X^2^ test, Unit: N (weighted %).

### Relationship Between Sociodemographic and Periodontal Disease

The prevalence of periodontal disease was 1.78 times higher among males compared with females (odds ratio [OR]: 1.78; 95% confidence interval, 95% CI: 1.63–1.95]) and 14.07 times higher among participants aged > 60 years compared to the 18–34 years groups (CI: 10.9–18.0). The prevalence of periodontal disease was 2.18 times higher among those who brush their teeth less than once a day compared to those who brush their teeth three times or more a day (CI: 1.88–2.53). The prevalence of periodontal disease was 2.75 times higher among those with diabetes (CI: 2.36–3.21).

### Relationship Between BMI or WC and the Presence of Periodontal Diseases

In terms of BMI, the risk of periodontal disease was 1.79 times higher among those with normal weight (CI: 1.95–3.33) and 2.55 times higher among the overweight compared to the underweight (CI: 1.38–2.33). In terms of WC, the risk of periodontal disease was 1.54 times higher among the obese (CI: 1.37–1.73) compared to the normal weight. However, no significant associations were observed after adjusting for confounders.

As a result of classifying BMI and WC into six groups, there was no subject in Group 2. In Group 1, where BMI was underweight but WC was normal, the prevalence of periodontal disease was the lowest (17.9%). The rate was the highest in Group 6 (39.0%), where BMI was high and WC was high.

Compared with Group 1, the risk for periodontal disease was 2.91 times higher in Group 6 (CI: 2.22–3.83) and 1.79 times higher after adjusting for the confounders (CI: 1.31–2.16) (Table 3).

**Table 3:** Relationship between sociodemographic and periodontal disease

Characteristics	Division	OR	CI
Gender	Female	1
Male	1.78	1.63–1.95
Age	19–34	1
35–59	2.21	1.92–2.32
≥ 60	14.07	10.98–18.05
Marital status	Married	1
Single	0.20	0.17–0.24
Education	≥ College	1
High	1.53	1.35–1.73
Middle	3.29	2.76–3.92
≤ Elementary	3.89	3.37–4.49
Income level	High	1
Middle–high	1.08	0.95–1.22
Middle–low	1.24	1.09–1.43
Low	1.39	1.19–1.63
Smoking	No	1
Yes	1.19	1.04–1.36
Alcohol use	No	1
Yes	0.67	0.59–0.77
Physical activity	Yes	1
No	1.47	1.33–1.62
Oral examination/ Within 1 year	Yes	1
No	1.41	1.28–1.56
Toothbrushing/day	≥ 3	1
2	1.41	1.28–1.55
≤ 1	2.18	1.88–2.53
Diabetes	No	1
Yes	2.75	2.36–3.21
Hypertension	No	1
Yes	1.57	1.60–2.33
Dyslipidaemia	No	1
Yes	1.48	1.33–1.64
By logistic regression analysis, Unit: N (weighted %). OR, odds ratio; 95% CI, 95% confidence intervals.

### Relationship Between BMI or WC and the Periodontal Diseases by Age

As a result of correlation between periodontal disease and BMI by age, the 35–59 years group risk for periodontal disease was 2.30 times higher in the overweight group compared to the underweight group (CI: 1.51–3.49) and after adjusting for the confounders the risk for periodontal disease was 1.61 times higher in the overweight than the underweight (CI: 1.01–2.56). As a result of correlation between periodontal disease and WC by age, of the 35–59 years group risk for periodontal disease was 1.61 times higher in the obese group compared with the normal group (CI: 1.39–1.87), and after adjusting for confounders group the risk for periodontal disease was 1.30 times higher (CI: 1.09–1.54) (Table 4).

**Table 4:** Odds ratio (95% confidence intervals) of body mass index and waist circumference with periodontal disease

Variable		Crude OR (95% CI)	^†^Adjusted OR (95% CI)
Group	Periodontal diseases	Crude OR (95% CI)	^†^Adjusted OR (95% CI)
N (%)
No	Yes
BMI	Underweight	1	1
	Normal	1.759 (1.954–3.337)	1.502 (0.273–8.278)
	High	2.554 (1.384–2.337)	1.429 (0.257–7.652)
WC	Normal	1	1
	High	1.547 (1.379–1.737)	1.177 (0.788–1.757)
Group 1^a^	375(82.1)	93(17.9)	1	1
Group 2^b^	0	0	0	0
Group 3^c^	5116(72.5)	1977(27.5)	1.73 (1.33–2.25)	1.32 (0.97–1.80)
Group 4^d^	336(61.3)	222(38.7)	2.88 (2.16–4.04)	1.19 (0.82–1.74)
Group 5^e^	868(71.8)	372(28.2)	1.79 (1.34–2.40)	1.34 (0.95–1.84)
Group 6^f^	1881(61.0)	1252(39.0)	2.91 (2.22–3.83)	1.79 (1.31–2.16)
By logistic regression analysis ^a^ Group 1: Underweight BMI and Normal WC, ^b^ Group 2: Underweight BMI and high WC (No subject in Group 2), ^c^ Group 3: normal BMI and normal WC, ^d^ Group 4: normal BMI, obese WC, ^e^ Group 5: high BMI, normal WC, ^f^ Group 6: high BMI and WC. ^†^Adjusted for gender, age, marital status, education, income level, smoking, alcohol use, physical activity, oral health examination, tooth brushing, diabetes, hypertension, dyslipidaemia. BMI, body mass index; WC, waist circumference; OR, odds ratio; CI, confidence intervals

## DISCUSSION

Periodontal disease is a common chronic disease in adults that affects oral health, nutritional intake, and the aesthetic aspects of an individual.^20^ Risk factors for periodontal diseases include age, sex, socioeconomic status, history of systemic diseases, obesity, and habits for maintaining oral health. Obesity results in many other chronic diseases, and the appropriate measurement of obesity may provide an additional evaluation of the risks of other diseases. In this study, the correlation between obesity and the risk of developing periodontal disease was assessed by combining two indicators, BMI and WC, to analyse its correlation to the prevalence of periodontal disease.

The study results showed that the rate of periodontal disease was higher when BMI and WC were obese, which was consistent with previous studies.^16,18^ The mechanism underlying the effects of obesity on periodontal diseases is considered to be attributed to the local and systemic effects of adipokines secreted by adipose tissues. Toxins such as leptin, tumour necrosis factor-α, adiponectin, angiotensinogen, and C-reactive peptide are estimated to induce inflammation in periodontal tissues.^12^ Moreover, the obese subjects tend to consume food with high sugar, and those with increased weight tend to be less concerned with maintaining oral health, which increases the risk for the development of periodontal disease.

After adjusting for confounding variables, the analysis revealed that higher BMI and WC were associated with an increased risk of periodontal disease in the 35–59 age group, consistent with previous studies.^1,16^ These findings suggest that, as individuals enter middle age, increased economic activity may lead to irregular lifestyle habits, such as overeating and excessive alcohol consumption, along with a systemic decline in metabolism and basal metabolic rate. Additionally, for women, the decrease in female hormones following menopause would have contributed to the results. However, there have also been studies reporting a higher risk of periodontal disease in younger age groups.^1,9^ Therefore, it is considered necessary to conduct further research that takes into account the age-specific characteristics.

According to the results of this study, the risk of periodontal disease was high in the obese group (Groups 4 and 6) with increased waist circumference. These results were similar to those of Kang.^14^ Also, a study by Park, which categorised BMI and WC into mild, moderate, and high obesity groups, found that the risk of intraocular pressure in the same WC group did not increase with higher BMI, while intraocular pressure in the same BMI group increased with higher WC.^25^ Although BMI has been regarded as a preferred indicator for assessing systemic risk and risks for developing periodontal disease, recent studies have suggested that WC is a more effective indicator for estimating disease occurrence. Since BMI does not account for body composition, such as distinguishing whether weight originates from fat or muscle, this study incorporated WC as an additional obesity indicator to specifically account for visceral fat. Abdominal fat tissue, as measured by WC, functions as an endocrine organ that secretes various proteins known as adipokines.^30^

This characteristic suggests a potential link between visceral fat accumulation and periodontal disease. Shimomura et al^28^ further identified visceral fat tissue as a key organ that secretes plasminogen activator inhibitor-1 (PAI-1), which plays a significant role in the pathogenesis of vascular diseases. Consequently, visceral fat accumulation has been proposed to exhibit a stronger correlation with disease risk compared to BMI due to its role in producing various inflammatory mediators. In light of these findings, it can be hypothesised that the elevated risk of periodontal disease observed in the group with obesity based on WC (Group 4/6) in this study may be closely associated with visceral fat accumulation. It is believed that the definition of obesity should consider both body fat mass and body fat distribution, and it is appropriate to incorporate more than two indicators of obesity in correlational studies.^14,15^

The relationship between obesity and periodontal diseases has been attributed to inflammatory mediators resulting from obesity, which aggravate periodontal diseases. In addition, neutrophil activation from periodontal diseases affects obesity.^15^ However, the exact mechanism by which obesity affects periodontal disease remains unknown and has not been sufficiently elucidated. The recent studies reporting that periodontal diseases interact with inflammatory substances such as cytokines show that inflammatory substances play a key role in connecting obesity and periodontal disease in a similar way to the interaction between obesity and metabolic diseases.^11^ A study by Wu et al, which compared the microbiota characteristics in obese and normal groups, demonstrated that the saliva in the obese group reduced the environmental adaptation capabilities of the microbials and the microbial degradation of non-biological components.^34^ In addition, a study by Goodson reported that the distribution of bacteria differs between obese and normal women. These reports suggest that inflammatory mediators alter the oral periodontal environment of an individual into an inflammatory state.^10^ Nevertheless, this cross-sectional analysis based on the KNHANES data is limited in its inability to explain a causal relationship. The limitation of this study pertains to the possibility of selection bias due to the nature of a cross-sectional study, including some participants of the oral examination and health survey. Also, the classification of obesity could differ among ethnicities for each indicator. However, this study is significant because it reclassified obesity indicators, BMI and WC, as a composite measure and analysed their association with periodontal disease. Future research is warranted to better understand the causal relationship between obesity and periodontal diseases and identify obesity indicators that signify this relationship.

In conclusion, the analysis of an association between periodontal diseases and obesity showed an increased risk for periodontal disease development in the two groups (Groups 4/6). The analysis of periodontal diseases in relation to obesity showed a high risk for periodontal diseases in the two groups with high WC (Groups 4/6).

Collectively, the results of the present study suggest that obesity is associated with periodontitis. In particular, the association between periodontal disease and obesity, as measured by BMI and WC, may be stronger in middle-aged adults (Table 5). Furthermore, when both indicators show obesity, it could serve as a significant risk factor for periodontal disease. Obesity is a substantial risk factor for periodontitis. Therefore, when evaluating periodontal risk factors, obesity indicators should be included. Also, to prevent periodontal disease, taking care of the bacterial plaque subgingivally and supragingivally, along with measures to prevent obesity, should be considered.

**Table 5:** Odds ratio (95% confidence intervals) of body mass index and waist circumference with periodontal disease stratified by age

Indicator of obesity	Age subgroups (years)
Crude OR (95% CI) ^†^Adjusted OR(95% CI)
19–34	35–59	≥ 60
**BMI**			
Underweight	1		
Normal	1.99 (0.74–5.31) ^†^ 1.66 (0.57–4.81)	1.57 (1.05–2.35) ^†^1.34 (0.86–2.08)	0.67 (0.56–1.45) ^†^ 1.03 (0.62–1.73)
High	2.99 (1.19–7.47) ^†^ 1.91 (0.75–5.24)	2.30 (1.51–3.49) ^†^1.61( 1.01–2.56)	0.67 (0.68–1.80) ^†^ 1.34 (0.81–2.26)
**WC**			
Normal	1		
High	1.764 (1.10–2.82) ^†^ 1.24 (0.73–2.09)	1.61 (1.39–1.87) ^†^ 1.30 (1.09–1.54)	1.18 (1.02–1.36) ^†^ 1.23 (1.05–1.44)
By Logistic regression analysis. ^†^Adjusted for gender, age, marital status, education, income level, smoking, alcohol use, physical activity, oral health examination, tooth brushing, diabetes, hypertension, dyslipidaemia. BMI, body mass index; WC, waist circumference; OR, odds ratio; CI, confidence intervals.
